# ﻿Ground spiders (Araneae, Gnaphosidae) from Jiangxi Province, China

**DOI:** 10.3897/zookeys.1108.85655

**Published:** 2022-06-24

**Authors:** Ke-Ke Liu, Jing Yan, Qi-xin Xiao, Chong Luo, Yong-hong Xiao, Alexander A. Fomichev

**Affiliations:** 1 College of Life Science, Jinggangshan University, Ji’an 343009, Jiangxi, China; 2 Altai State University, Lenina Pr., 61, Barnaul, RF-656049, Russia; 3 Tomsk State University, Lenina Pr., 36, Tomsk, RF-634050, Russia; 4 Key Laboratory of Agricultural Environmental Pollution Prevention and Control in Red Soil Hilly Region of Jiangxi Province, Jinggangshan University, Ji’an, 343009, Jiangxi, China

**Keywords:** Asia, biodiversity, distribution, gnaphosid spiders, new species, taxonomy

## Abstract

A list of 26 gnaphosid species belonging to 14 genera collected in Jiangxi Province, China, is provided. Three new species of ground spiders from Jiangxi Province of China are diagnosed, described, and illustrated: *Haplodrassusyinae* Liu, **sp. nov.** (♂♀), *Hitobiaxiaoxi* Liu, **sp. nov.** (♂), and *Zelotesdingnan* Liu, **sp. nov.** (♂♀). *Haplodrassusyinae* Liu, **sp. nov.** was previously erroneously recorded in Jiangxi Province as *H.montanus*Yin et al., 2012.

## ﻿Introduction

The rich biodiversity of China is likely due to two reasons: cenozoic tectonic evolution in the Tethyan, has greatly changed the landforms and environment of Eurasia and driven the evolution of animals (Zhao et al. 2022), and China’s mountainous landscape has provided refuge for organisms, enabling species such as spiders to survive glacial periods (Yao et al. 2021; Lu et al. 2022). Approximately 5700 spider species from 69 families have been recorded from China (Biodiversity Committee of Chinese Academy of Sciences 2022). The southwest (Himalayan and Hengduan mountains, and Yunnan-Guizhou Plateau), has been the center of diversification for several spider groups (Li 2020) and has become a biodiversity hotspot for researchers. Many spider species are discovered from there in the last 15 years (Li and Lin 2016; Li 2020). In contrast to these areas, species richness of spiders in non-hotspots, such as Jiangxi Province, has not been given enough attention. Recently, in Jiangxi, which has a mountain terrain, many spider taxa have been discovered, such as Agelenidae (Liu et al. 2020a, 2021a), Dictynidae (Liu et al. 2018), Oonopidae (Liu et al. 2016, 2019), Phrurolithidae (Liu et al. 2020b, 2020c, 2021b, 2022a), Salticidae (Liu et al. 2017b, 2022c), Thomisidae (Liu et al. 2017a, 2022b), and Gnaphosidae (this study). These discoveries lend support to mountainous landscapes being refuges for spiders during glacial periods.

Gnaphosidae Banks, 1892, commonly known as ground spiders, is the sixth largest spider family with a global distribution, comprising 2414 extant species belonging to 144 genera (WSC 2022). Currently, 213 species belonging to 36 genera are known from China (Li and Lin 2016; Li 2020). Although Gnaphosidae is the ninth largest family in China, the number of taxonomic papers dealing with this family in the last decade is few (WSC 2022). Among species of ground spiders recorded from China, more than 60% are recorded from northern China, namely from Qinghai, Xinjiang, Neimenggu, Hebei, Gansu, Beijing, Shanxi, Liaoning, Xizang, and Henan provinces. The family is poorly studied in Jiangxi Province located in southern China, and only four species of Gnaphosidae are recorded from this province: *Allozeloteslushan* Yin & Peng, 1998, *Gnaphosakompirensis* Bösenberg & Strand, 1906, *Hitobiayasunosukei* Kamura, 1992, and *Zelotesliaoi* Platnick & Song, 1986 (Li and Lin 2016).

While studying ground spiders from Jiangxi Province, we came across several undescribed and poorly known species, as well as many described species. The aims of the present paper are to provide detailed descriptions of three new species and to report findings of 26 species belonging to 14 genera.

## ﻿Materials and methods

Specimens were examined using a Zeiss Stereo Discovery V12 stereomicroscope with a Zoom Microscope System. Both male palps and female copulatory organs were detached and examined in 80% ethanol, using a Zeiss Axio Scope A1 compound microscope with a KUY NICE CCD. The epigynes were cleared in trypsin enzyme solution to dissolve soft tissues. For SEM photographs, specimens were dried under natural conditions, sprayed with gold with a small ion-sputtering apparatus ETD-2000, or left without coating, and photographed with a ZEISS EVO LS15 scanning electron microscope. Specimens, including detached male palps and epigynes, were stored in 75% ethanol after examination. All the specimens are deposited in Animal Specimen Museum, Life Science of College, Jinggangshan University (**ASM-JGSU**).

Measurements were taken with the AxioVision software (SE64 rel. 4.8.3) and given in millimetres. Terminology of the male and female copulatory organs follows Platnick and Shadab (1982) and Fomichev and Marusik (2021). Leg measurements are given as total length (femur, patella, tibia, metatarsus, tarsus).

### ﻿The abbreviations used in the text are as follows

**Eyes: ALE**, anterior lateral eye; **AME**, anterior median eye; **MOA**, median ocular area; **PLE**, posterior lateral eye; **PME**, posterior median eye.

**Leg segments: Fe**, femur; **Mt**, metatarsus;**Pt**, patella; **Ta**, tarsus; **Ti**, tibia.

**Spination: d**, dorsal; **p**, prolateral; **r**, retrolateral; **v**, ventral.

**Male palp: BP**, basal process; **Co**, conductor; **EA**, embolic apophysis; **Em**, embolus; **EP**, embolic projection; **IS**, intercalary sclerite; **LaP**, lamellar process; **MA**, median apophysis; **RTA**, retrolateral tibial apophysis; **SD**, sperm duct; **StP**, subterminal process; **TA**, terminal apophysis; **TP**, terminal process; **UP**, upper process.

**Epigyne: AP**, anterior pocket; **CD**, copulatory duct; **CO**, copulatory opening; **FD**, fertilization ducts; **Fo**, fovea; **H**, hood; **LG**, lateral gland; **MP**, median pocket; **PP**, posterior pocket; **Se**, septum; **Sp**, spermatheca.

## ﻿Taxonomic survey

### Family Gnaphosidae Banks, 1892

The known gnaphosid spider fauna of Jiangxi Province is complemented by 23 additional species belonging to 13 genera and now numbers 27 species in 14 genera. The full list of gnaphosid spiders recorded in this province is presented in Table 1.

**Table 1.** List of Gnaphosidae species recorded in Jiangxi Province. Genera recorded for the first time are marked with an asterisk (*).

Genus	Species	No. of ♂♂	No. of ♀♀	Total
*Allozelotes* Yin & Peng, 1998	*A.lushan* Yin & Peng, 1998	0	1	1
*Aphantaulax* Simon, 1878 *	*A.trifasciata* (O. Pickard-Cambridge,1872)	0	1	1
*Cladothela* Kishida, 1928 *	*C.oculinotata* (Bösenberg & Strand, 1906)	1	0	1
	*C.parva* Kamura, 1991	1	1	2
*Drassyllus* Chamberlin, 1922 *	*Drassyllus* sp. 1	0	13	13
	*D.sanmenensis* Platnick & Song, 1986	14	18	32
	*Drassyllus* sp. 2	0	7	7
*Gnaphosa* Latreille, 1804	*G.hastata* Fox, 1937	0	1	1
	*G.kompirensis* Bösenberg & Strand, 1906	1	1	2
*Haplodrassus* Chamberlin, 1922 *	*H.yinae* sp. nov.	1	3	4
*Hitobia* Kamura, 1992	*H.taiwanica* Zhang, Zhu & Tso, 2009	1	0	1
	*H.xiaoxi* sp. nov.	1	0	1
	*H.yasunosukei* Kamura 1992	2	0	2
*Odontodrassus* Jézéquel, 1965 *	*O.hondoensis* (Saito, 1939)	1	0	1
*Pseudodrassus* Caporiacco, 1935 *	*P.pichoni* Schenkel, 1963	0	1	1
*Sanitubius* Kamura, 2001 *	*S.anatolicus* (Kamura, 1989)	0	1	1
*Scotophaeus* Simon, 1893 *	*S.hunan* Zhang, Song & Zhu, 2003	1	1	2
*Sernokorba* Kamura, 1992 *	*S.fanjing* Song, Zhu & Zhang, 2004	0	2	2
	*S.pallidipatellis* Bsenberg & Strand, 1906	2	0	2
*Synaphosus* Platnick & Shadab, 1980 *	*S.daweiensis* Yin, Bao & Peng, 2002	5	2	7
*Zelotes* Gistel, 1848	*Z.asiaticus* (Bösenberg & Strand, 1906)	12	4	16
	*Z.dingnan* sp. nov.	4	1	5
	*Z.potanini* Schenkel, 1963	1	0	1
	*Z.sanmen* Platnick & Song, 1986	0	6	6
	*Z.wuchangensis* Schenkel, 1963	2	0	2
	*Z.yinae* Platnicket & Song, 1986	0	1	1

#### ﻿Genus Haplodrassus

Taxon classificationAnimaliaAraneaeGnaphosidae

Chamberlin, 1922

ED84DC85-289C-5555-BCCA-F4E499D1D1A3

##### Comments.

This genus includes 83 species, mainly distributed in the Palaearctic (WSC 2022). A smaller number of species are known from the Nearctic and Oriental realms (WSC 2022). The genus was divided into nine species groups, based on morphological characteristics, by Omelko and Marusik (2012), i.e., the *caspius*, *dalmatensis*, *kulczynskii*, *mediterraneus*, *montanus*, *signifier*, *silvestris*, *tegulatus*, and *umbratilis* groups. To date, only 13 species of *Haplodrassus* are recorded from China. Except for *Haplodrassusguiyangensis* Yan & Yu, 2021, the others were recorded more than 10 years ago.

#### ﻿ Haplodrassus yinae

Taxon classificationAnimaliaAraneaeGnaphosidae

Liu sp. nov.

F957AC71-2FCC-562B-B5AE-842B3218E5F3

https://zoobank.org/A9BEB099-2D07-4226-B5BF-95D11DD643AC

Figs 1 , 2 , 3

Haplodrassus montanus Yin et al., 2012: 1177, fig. 627a–f (♂♀).

##### Material examined.

***Holotype*** ♂, China: Jiangxi Province, Ji’an City, Jinggangshan County Level City, Jinggang Mountain National Nature Reserve, Dongshang Town, Jiangshan Village, 26°46'01.56"N, 113°54'53.65"E, 326 m, 4.II.2021, K. Liu et al. leg. ***Paratypes***: 2 ♂, 1 ♀, the same data as the holotype.

##### Etymology.

The specific name is a matronym in honour of Prof. Changmin Yin, the first to find and recognise this species, in honour of her great contribution to Chinese arachnology; noun (name) in genitive case.

##### Diagnosis.

**Figure 1.**
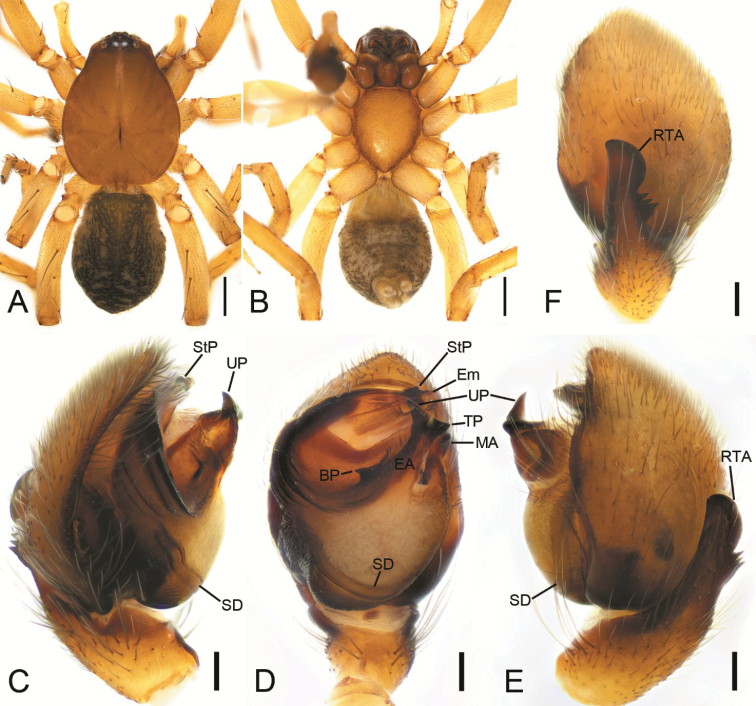
*Haplodrassusyinae* sp. nov., male holotype **A** habitus, dorsal view **B** same, ventral view **C** palp, prolateral view **D** same, ventral view **E** same, retrolateral view **F** same, dorsal view. Abbreviations: BP – basal process, EA – embolic apophysis, Em – embolus, MA – median apophysis, RTA – retrolateral tibial apophysis, SD – sperm duct, StP – subterminal process, TP – terminal process, UP – upper process. Scale bars: 0.5 mm (**A, B**); 0.1 mm (**C–F**).

**Figure 2.**
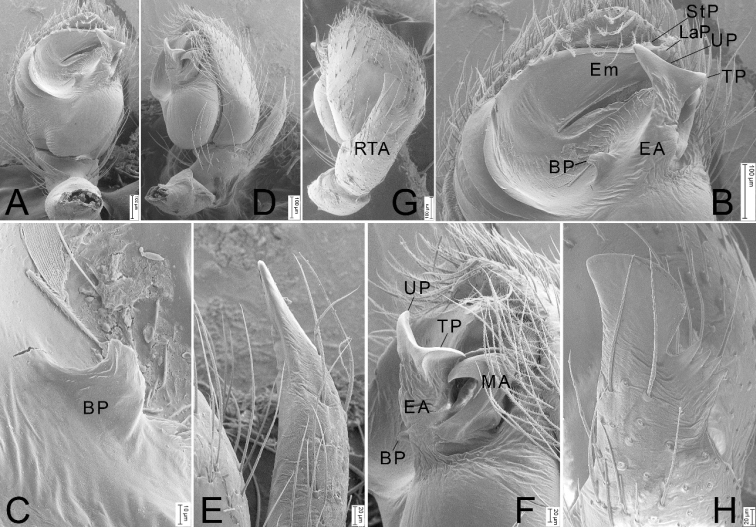
SEM micrographs of *Haplodrassusyinae* sp. nov., male palp (paratype) **A** ventral view **B** same, detail of embolic division **C** same, details of basal process of embolic base **D** retrolateral view **E** same, details of RTA**F** same, detail of embolic division **G** dorsal view, slightly retrolaterally **H** same, details of RTA. Abbreviations: BP – basal process, EA – embolic apophysis, Em – embolus, LaP – lamellar process, MA – median apophysis, RTA – retrolateral tibial apophysis, StP – subterminal process, TP – terminal process, UP – upper process.

**Figure 3.**
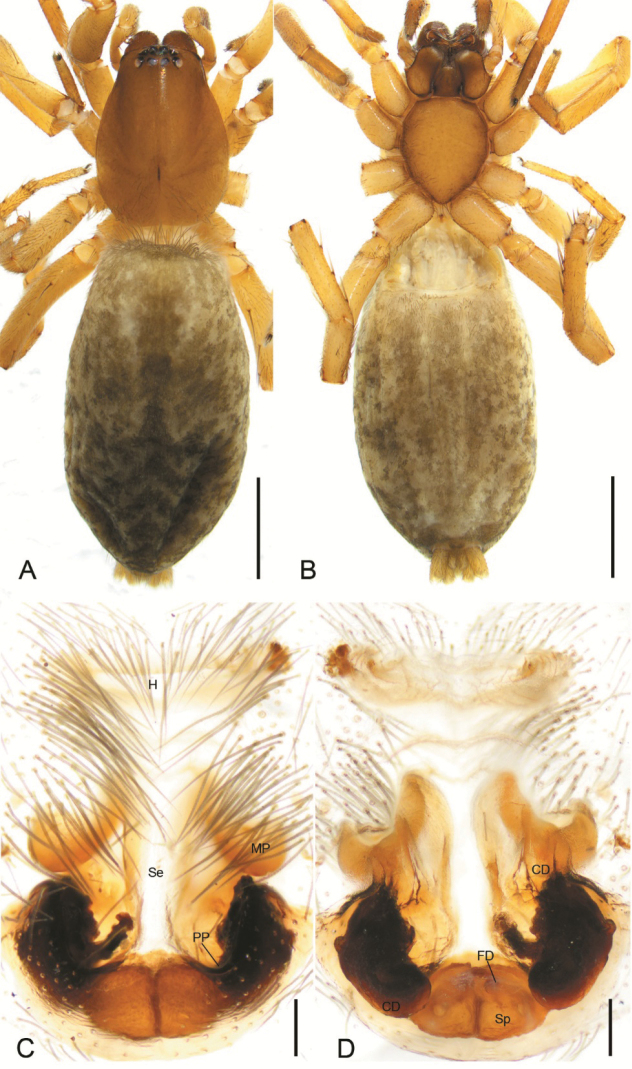
*Haplodrassusyinae* sp. nov., female paratype **A** habitus, dorsal view **B** same, ventral view **C** epigyne, ventral view **D** same, dorsal view. Abbreviations: CD – copulatory duct, FD – fertilization duct, H – anterior hood, MP – median pocket, PP – posterior pocket, Se – septum, Sp – spermatheca. Scale bars: 0.5 mm (**A, B**); 0.1 mm (**C, D**).

The new species belongs to the *montanus* group. The male of the new species is similar to *H.guiyangensis* Yan & Yu, 2021, *H.hatsushibai* Kamura, 2007, *H.huarong* Yin & Bao, 2012, and *H.montanus* Paik & Sohn, 1984 in having an oval tegulum, a bifurcate embolic apophysis (EA), and a hook-shaped median apophysis (MA), but it can be differentiated from *H.hatsushibai* and *H.montanus* by the absence of the basal tooth on the embolus (cf. Figs 1D, 2A, B vs Omelko and Marusik 2012: figs 7, 10). In addition, *H.yinae* sp. nov. possess 5 or 6 ridges on the embolic base (vs 6–8 ridges in *H.guiyangensis*; 7 or 8 ridges in *H.hatsushibai*; 6 or 7 ridges in *H.huarong*; 3 or 4 ridges in *H.montanus*) (cf. Fig. 1C, D vs Yan et al. 2021: figs 1A, C, 2A, B and Omelko and Marusik 2012: figs 7, 8, 10, 11) and has a small tooth-like basal process (BP) directed at 9 o’clock in ventral view (vs 11 o’clock in *H.guiyangensis*; a large laminar, tooth-like basal process, directed at 11 o’clock position in *H.hatsushibai*, *H.huarong* and *H.montanus*) (cf. Figs 1D, 2A, B vs Yan et al. 2021: figs 1C, 2B and Omelko and Marusik 2012: figs 7, 10 and Yin et al. 2012: fig. 625f, g). The female of the new species resembles those of *H.montanus* in having pair of posterior pockets (PP) located in posterolateral part of the atrium, but it can be distinguished by the septum (Se) narrowing posteriorly (vs septum narrowing anteriorly) (cf. Fig. 3C vs Omelko and Marusik 2012: fig. 20). Also, it can be separated from *H.hatsushibai* by posterior pockets located in posterolateral part of the atrium (vs posterior pockets located in posteromedial part of the atrium) (cf. Fig. 3C vs Omelko and Marusik 2012: fig. 23) and from *H.huarong* by the copulatory ducts (CD) as wide as spermathecae (Sp) (vs 1/3 of spermathecal width) (cf. Fig. 3D and Yin et al. 2012: fig. 625e).

##### Description.

**Male.** Habitus as in Fig. 1A, B. Total length 3.65. Carapace: 1.89 long, 1.51 wide. Carapace covered with a few strong setae. Eye sizes and interdistances: AME 0.08, ALE 0.11, PME 0.09, PLE 0.10, AME–AME 0.07, AME–ALE 0.03, PME–PME 0.02, PME–PLE 0.05, AME–PME 0.08, AME–PLE 0.12, ALE–ALE 0.24, PLE–PLE 0.33, ALE–PLE 0.04. MOA 0.21 long, front width 0.20, back width 0.23. Chelicera with 4 promarginal and 2 retromarginal teeth. Abdomen: 1.77 long, 1.21 wide. Leg measurements: I 4.08 (1.1, 0.57, 1.08, 0.69, 0.64); II 3.72 (1.11, 0.62, 0.84, 0.59, 0.56); III 3.06 (0.97, 0.4, 0.56, 0.62, 0.51); IV 4.91 (1.4, 0.58, 1.14, 1.17, 0.62). Leg spination: I Fe: d2, p1; Mt: p1; II Fe: d2; Pa: r1; Ti: v2; III Fe: d2, p1, r1; Ti: d1, p2, r2, v6; Mt: p3, r3, v4; IV: Fe: d2, r1; Pa: r1; Ti: p2, r3, v6; Mt: p2, r2, v6.

Colouration (Fig. 1A, B). Carapace, chelicerae and sternum brown. Maxillae and labium reddish brown. Legs yellow. Palps yellow, cymbium brown. Abdomen dark brown, with two pairs of longitudinal yellowish markings anteromedially and four pairs of chevrons posteromedially. Spinnerets yellow.

Palp as in Figs 1C–F, 2. RTA thumb-shaped, 2× longer than tibia, bearing three strong teeth. Cymbium 2× longer than wide. Tegulum ovate, with a distinct depression in the anterior part. Sperm duct (SD) stretched along the posterior margin of the tegulum. Embolic apophysis (EA) with three well developed processes: the basal one (BP) hook-shaped and small, directed at 9 o’clock; bases of terminal (TP) and upper processes (UP) touching each other together, forming a fishtail-shape. Upper process and terminal process triangular, approximately the same length. Median apophysis (MA) hook-shaped, twice shorter than embolic apophysis. Embolic base with five or six ridges prolaterally. Apex of embolus bears subterminal process (StP) and lamellar process (LaP).

**Female.** Habitus as in Fig. 3A, B. Total length 5.13. Carapace: 1.8 long, 1.33 wide. Eye sizes and interdistances: AME 0.08, ALE 0.11, PME 0.08, PLE 0.10, AME–AME 0.07, AME–ALE 0.02, PME–PME 0.03, PME–PLE 0.06, AME–PME 0.08, AME–PLE 0.13, ALE–ALE 0.22, PLE–PLE 0.34, ALE–PLE 0.06. MOA 0.24 long, front width 0.19, back width 0.22. Chelicera with 4 promarginal and 2 retromarginal teeth. Abdomen: 3.19 long, 1.86 wide. Leg measurements: I 2.9 (0.88, 0.45, 0.67, 0.44, 0.46); II 3.2 (0.95, 0.49, 0.72, 0.51, 0.53); III 2.49 (0.95, 0.32, 0.41, 0.42, 0.39); IV 3.26 (0.89, 0.43, 0.68, 0.75, 0.51). Leg spination: I Fe: d2, p1; Mt: p1; II Fe: d2; Pa: r1 Ti: v2; III Fe: d2, p1, r1; Ti: d1 p2, r2, v6; Mt: p3, r3, v4; IV: Fe: d2, r1; Pa: r1; Ti: p2, r3, v6; Mt: p2, r2, v6.

Epigyne as in Fig. 3C, D. Epigyne 1.3× longer than width. Anterior hood (H) flat, 7× wider than long. Septum (Se) expands anteriorly. Median pockets (MP) concave backwards. Posterior pockets deep, located at posterolateral part of the atrium. Copulatory openings invisible, arising from median pockets and covered by the margin of median pockets. Copulatory ducts (CD) nearly 3× longer than width. Spermathecae (Sp) stuck together, as long as wide. Fertilisation ducts (FD) directed laterally.

##### Distribution.

China: Jiangxi and Hunan provinces (Fig. 9).

#### ﻿Genus Hitobia

Taxon classificationAnimaliaAraneaeGnaphosidae

Kamura, 1992

5E773681-6B44-54A6-B7DA-82D50459FA8E

##### Comments.

The genus includes 21 species, all of which are distributed in south, south-east, or east Asia (China, Thailand, Korea, Japan, Vietnam, India) (WSC 2022). More than two-thirds of all known species have been described and/or reported from China.

#### ﻿ Hitobia xiaoxi

Taxon classificationAnimaliaAraneaeGnaphosidae

Liu sp. nov.

6BA7571F-8FBC-58B2-A59B-00BE7D87A068

https://zoobank.org/1183FC4C-27BB-41CE-AA87-3B85437FE1D4

Figs 4 , 5

##### Material examined.

***Holotype***: ♂, China: Jiangxi Province, Ji’an City, Jinggangshan County Level City, Jinggang Mountain National Nature Reserve, Huangao Town, Fuxi Village, Xiaoxi Forest Farm, 26°28'22.92"N, 114°11'53.07"E, 413 m, 14.XI.2020, Liu et al. leg.

##### Etymology.

The specific name derived from the type locality is a noun in apposition.

##### Diagnosis.

**Figure 4.**
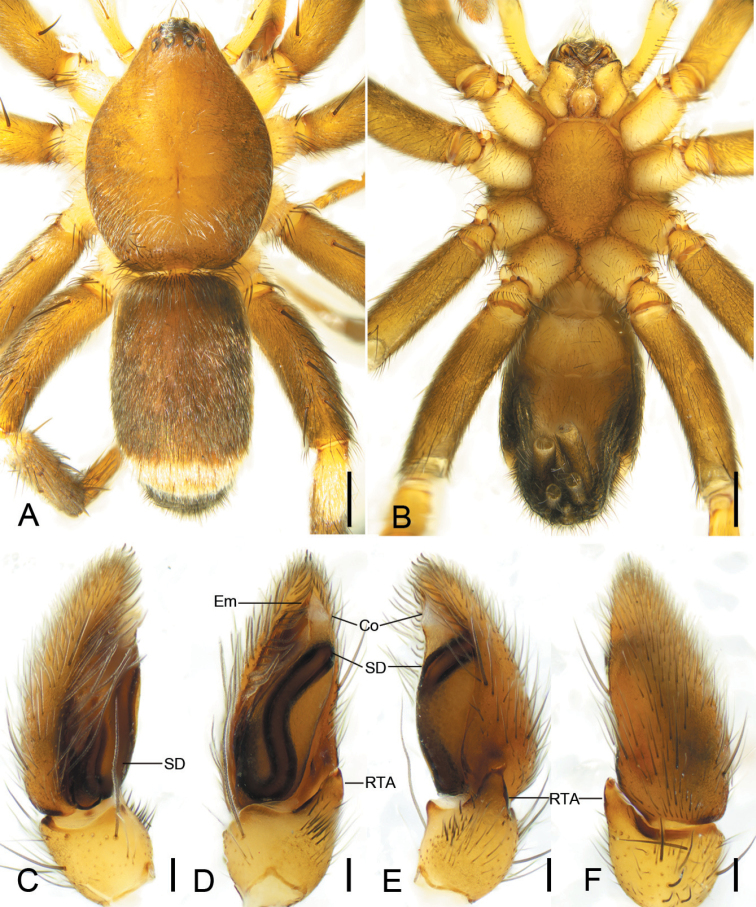
*Hitobiaxiaoxi* sp. nov., male holotype **A** habitus, dorsal view **B** same, ventral view **C** palp, prolateral view **D** same, ventral view **E** same, retrolateral view **F** same, dorsal view. Abbreviations: Co – conductor, Em – embolus, RTA – retrolateral tibial apophysis, SD – sperm duct. Scale bars: 0.5 mm (**A, B**); 0.1 mm (**C–F**).

**Figure 5.**
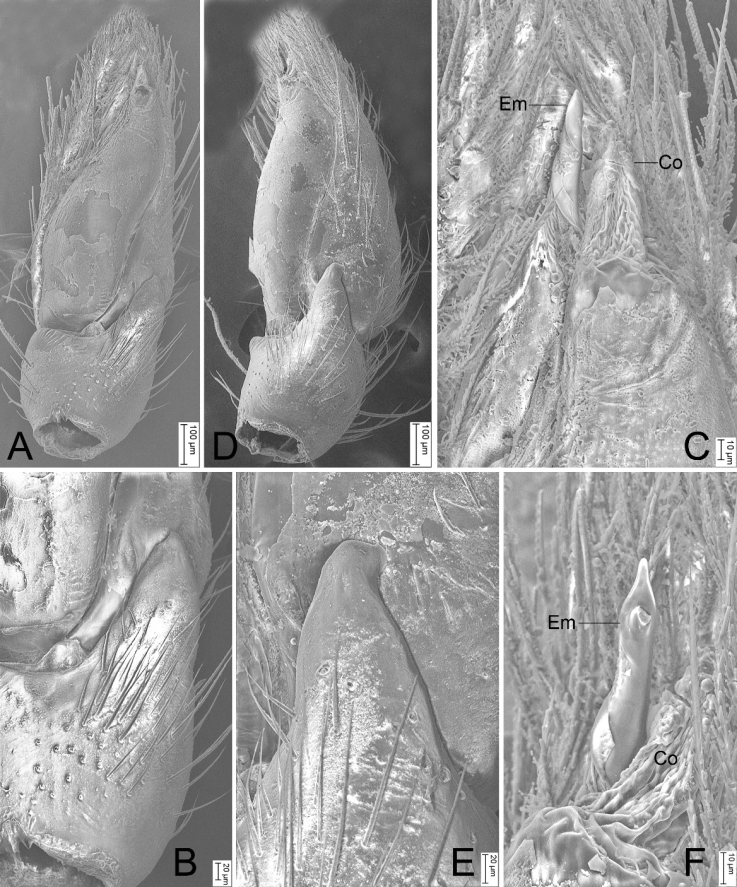
SEM micrographs of *Hitobiaxiaoxi* sp. nov., male palp (holotype) **A** ventral view **B** same, details of RTA**C** same, detail of embolic division **D** retrolateral view **E** same, details of RTA**F** same, detail of embolic division. Abbreviations: Co – conductor, Em – embolus.

The male of this new species is similar to *Hitobiashaohai* Yin & Bao, 2012 and *H.taiwanica* Zhang, Zhu & Tso, 2009 in having a short RTA and retrolaterally oriented embolus (Em), but it differs from them in the subtriangular RTA (vs beak-like in *H.shaohai* and *H.taiwanica*) and the embolus with twisted apex (vs twisted apex absent in *H.shaohai* and *H.taiwanica*) (cf. Figs 4E, 5C–F vs Yin et al. 2012: fig. 631d and Zhang et al. 2009: fig. 1E, F).

##### Description.

**Male.** Habitus as in Fig. 4A, B. Total length 4.97. Carapace: 2.18 long, 1.74 wide. Eye sizes and interdistances: AME 0.09, ALE 0.09, PME 0.08, PLE 0.08, AME–AME 0.06, AME–ALE 0.21, PME–PME 0.1, PME–PLE 0.07, AME–PME 0.12, AME–PLE 0.21, ALE–ALE 0.23, PLE–PLE 0.42, ALE–PLE 0.12. MOA 0.3 long, front width 0.2, back width 0.27. Chelicera with 2 promarginal and 1 retromarginal teeth. Abdomen: 2.64 long, 1.29 wide. Abdomen covered with numerous setae dorsally. Scutum covers more than 2/3 of abdomen. Leg measurements: I 5.09 (1.47, 0.71, 1.21, 1.01, 0.69); II 4.99 (1.47, 0.68, 1.13, 0.98, 0.73); III 5.15 (1.37, 0.74, 1.05, 1.27, 0.72); IV 6.74 (1.78, 0.93, 1.46, 1.91, 0.66). Leg spination: I Fe: d3, p2; Pa: r1; Ti: p2, v6; Mt: v2; II Fe: d3, p2, r1; Ti: p2, v5; Mt: v2; III Fe: d3, p2, r2; Pa: d1, p1, r1; Ti: d1, p3, r2, v5; Mt: d2, p3, r3, v2; IV: Fe: d2, p1, r1; Pa: p1, r1; Ti: p3, r2, v5; Mt: d2, p3, r3, v6.

Colouration (Fig. 4A, B). Carapace, sternum, chelicerae, labium, and maxillae yellow-brown. Legs brown. Palps yellow, cymbium brown. Abdomen dark brown, with one transverse white stripe posteriorly. Spinnerets yellow grey.

Palp as in Figs 4C–F, 5. Femur with three strong spines dorsally. Patella with single spine dorsally. RTA subtriangular, slightly shorter than tibia, apex directed dorsally. Cymbium 2.5× longer than wide. Tegulum simple and smooth, tapers anteriorly. Conductor (Co) membranous. Sperm duct (SD) U-shaped. Embolus longer than conductor (Em), cone-shaped and twisted.

**Female.** Unknown.

##### Comments.

The new species together with *H.shaohai* and *H.taiwanica* clearly belongs to the same group based on configuration of their male palps. Unfortunately, only one male of *H.xiaoxi* sp. nov. was found and several *Hitobia* species from southern China are known only from females: *H.chayuensis* Song, Zhu & Zhang, 2004, *H.shimen* Yin & Bao, 2012, and *H.yunnan* Song, Zhu & Zhang, 2004. Thus, *H.xiaoxi* sp. nov. may be a junior synonym of any of the above-mentioned species. However, it should be noted that the new species differs from all females by the abdominal pattern of a broad arc-shaped white stripe subposteriorly (vs wavy wite stripe in *H.yunnan*, thin transverse white stripe in *H.chayuensis*, and herringbone-pattern in *H.shimen*). For this reason, we consider *H.xiaoxi* sp. nov. as a separate species. This hypothesis will be confirmed or rejected in the future when both sexes of the new species are collected together.

##### Distribution.

Known only from the type locality, Jiangxi Province, China (Fig. 9).

#### ﻿Genus Zelotes

Taxon classificationAnimaliaAraneaeGnaphosidae

Gistel, 1848

AE9B4913-3623-5EA9-9D9B-271E8166111A

##### Comments.

With 397 described species and worldwide distribution, *Zelotes* is the most speciose genus of the family (WSC 2022).

#### ﻿ Zelotes dingnan

Taxon classificationAnimaliaAraneaeGnaphosidae

Liu sp. nov.

61354E98-314E-5BA8-8DF7-8622CD5CBDAB

https://zoobank.org/AA3583D1-87C9-4B52-9983-04AFACAF5E40

Figs 6 , 7 , 8

##### Material examined.

***Holotype***: ♂, China: Jiangxi Province, Ganzhou City, Dingnan County, Lingbei Town, Aonao Village, near 42^#^ poles, 25°01'48.95"N, 115°06'11.01"E, 395 m, 5.X.2020, K. Liu et al. leg. ***Paratype***: 1 ♂: Dayu County, Neiliang Town, Tianhua Mountain, 25°25'38.09"N, 114°01'43.95"E, 1019 m, 3.X.2020, other data same as holotype; 1 ♂: near the county boundary between Xunwu and Anyuan Counties, Guizhumao parking lot, 24°55'35.36"N, 115°27'25.09"E, 716 m, 7.X.2020, other data same as holotype; 1 ♀: Anyuan county, Sanbaishan National Forest Park, hiking trails, 25°00'28.19"N, 115°25'59.45"E, 511 m, other data as same as previous; 1 ♂: Ji’an City, Jinggangshan Level City, Dongshang Town, Jiangshan Village, Qilichuan, 26°46'88.81"N, 113°52'00.83"E, 665 m, 4.II.2021, K. Liu et al. leg.

##### Etymology.

The specific name derived from the county where the type locality is located and is a noun in apposition.

##### Diagnosis.

**Figure 6.**
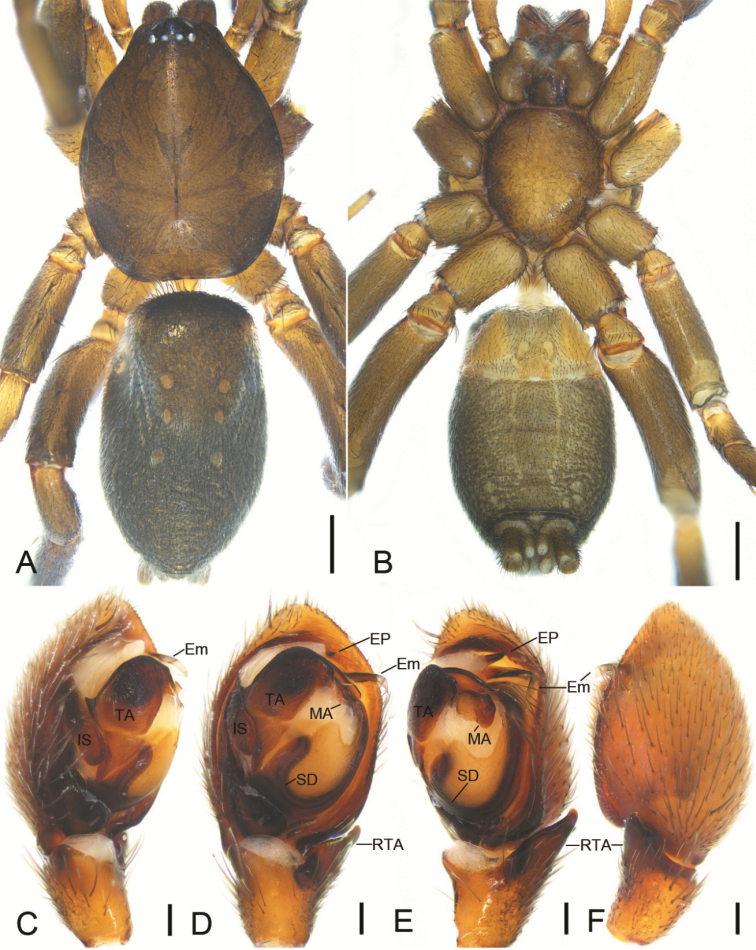
*Zelotesdingnan* sp. nov., male holotype **A** habitus, dorsal view **B** same, ventral view **C** palp, prolateral view **D** same, ventral view **E** same, retrolateral view **F** same, dorsal view. Abbreviations: Em – embolus, EP – embolic projection, IS – intercalary sclerite, MA – median apophysis, RTA – retrolateral tibial apophysis, SD – sperm duct, TA – terminal apophysis. Scale bars: 0.5 mm (**A, B**); 0.1 mm (**C–F**).

**Figure 7.**
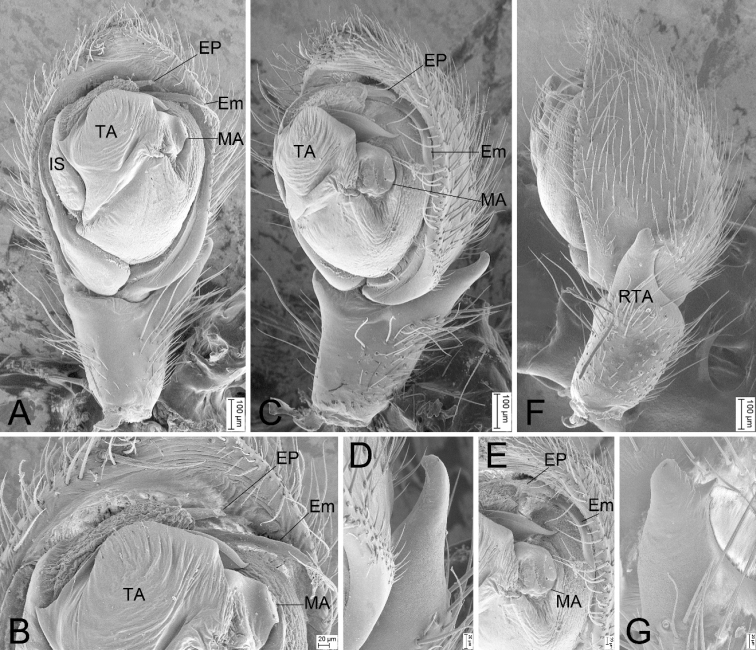
SEM micrographs of *Zelotesdingnan* sp. nov., male palp (paratype) **A** ventral view **B** same, details of embolic division **C** retrolateral view, slightly ventrally **D** same, details of RTA**E** same, details of embolic division **F** dorsal view, slightly retrolaterally **G** same, details of RTA. Abbreviations: Em – embolus, EP – embolic projection, IS – intercalary sclerite, MA – median apophysis, RTA – retrolateral tibial apophysis, TA – terminal apophysis.

**Figure 8.**
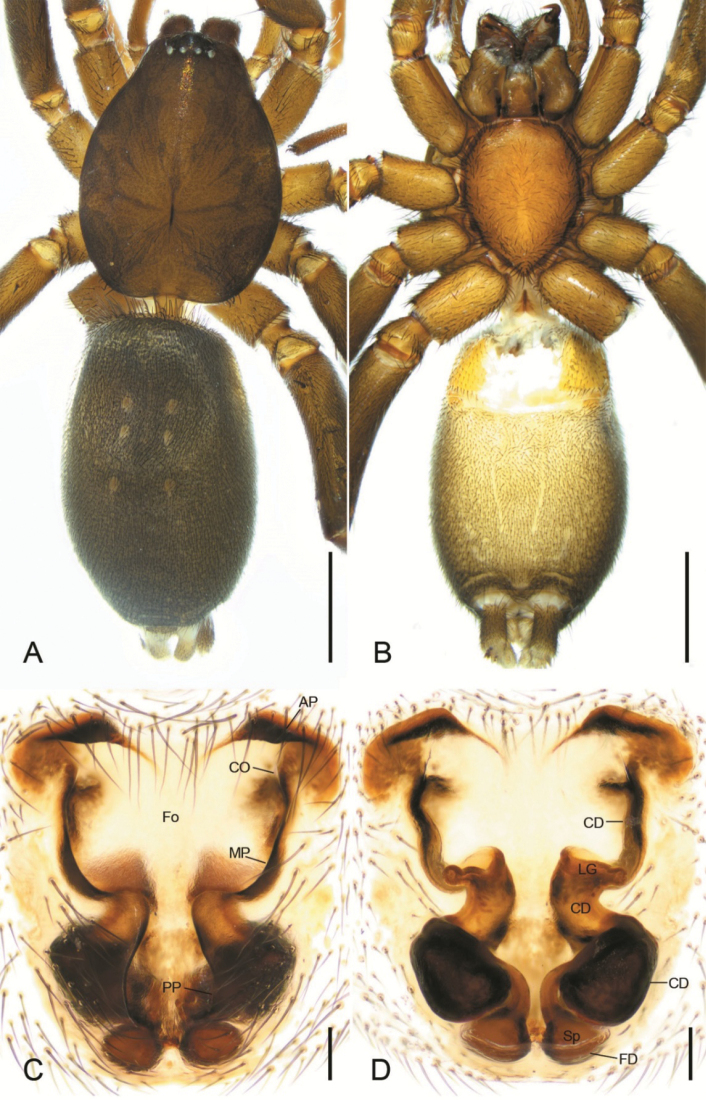
*Zelotesdingnan* sp. nov., female paratype **A** habitus, dorsal view **B** same, ventral view **C** epigyne, ventral view **D** same, dorsal view. Abbreviations: AP – anterior pocket, CD – copulatory duct, CO – copulatory opening, FD – fertilization duct, Fo – fovea, LG – lateral gland, MP – median pocket, PP – posterior pocket, Sp – spermatheca. Scale bars: 0.5 mm (**A, B**); 0.1 mm (**C, D**).

The male of this new species is similar to *Zelotesliaoi* Platnick & Song, 1986 in having a finger-like RTA, U-shaped sperm duct (SD), a triangular median apophysis (MA) in ventral view and a strong terminal apophysis (TA) with a spine-like apex, but differs by the clearly visible embolic projection (EP) (vs invisible or absent) and by the embolus (Em) with a membranous apex bending toward posterior part of tegulum (vs sclerotized apex bending toward anterior part of tegulum) (Figs 6C–F, 7 vs Song et al. 2004: fig. 158C, D). The female of *Z.dingnan* can be distinguished from that of *Z.liaoi* by the teardrop posterior part of the fovea (Fo) (vs subtriangular) and by the copulatory ducts (CD) divided into three parts (anterior, medial, and posterior) (vs two) (Figs 8C, D vs Song et al. 2004: fig. 158A, B).

##### Description.

**Male.** Habitus as in Fig. 6A, B. Total length 4.81. Carapace: 2.23 long, 1.75 wide. Eye sizes and interdistances: AME 0.06, ALE 0.09, PME 0.07, PLE 0.08, AME–AME 0.03, AME–ALE 0.03, PME–PME 0.04, PME–PLE 0.04, AME–PME 0.11, AME–PLE 0.12, ALE–ALE 0.17, PLE–PLE 0.23, ALE–PLE 0.02. MOA 0.23 long, front width 0.18, back width 0.19. Chelicera with 3 promarginal and 4 retromarginal teeth. Abdomen: 2.48 long, 1.5 wide. Leg measurements: I 6.04 (1.57, 0.89, 1.37, 1.16, 1.05); II 4.89 (1.39, 0.6, 1.12, 0.94, 0.84); III 5.2 (0.95, 0.68, 1.36, 1.51, 0.7); IV 6.43 (1.77, 0.7, 1.51, 1.57, 0.88). Leg spination: I Fe: d3; II Fe: d2, r1; Ti: v1; Mt: v4; III Fe: d2, p1, r2; Pa: r1; Ti: p2, r2, v5; IV: Fe: d2, r1; Pa: r1; Ti: p2, r1, v6; Mt: d6, p2, r3, v4.

Colouration (Fig. 6A, B). Carapace dark yellow-brown, with radial dark stripes dorsally. Chelicerae, maxillae, labium and sternum dark yellow-brown. Legs brown. Palps yellow-brown. Abdomen dark brown. Spinnerets brown.

Palp as in Figs 6C–F, 7. RTA finger-like, slightly longer than tibia in retrolateral view, apex directed dorsally. Apex of RTA bears two spines. Cymbium 2× longer than wide. Tegulum elliptical. Sperm duct (SD) U-shaped, originating from near median apophysis. Median apophysis (MA) with a broad base and retrolaterally curved apex. Terminal apophysis (TA) subquadrangular, with a tooth-like apophysis prolaterally and a spine-like apophysis anteriorly. Intercalary sclerite (IS) longer than terminal apophysis, with membranous part anteriorly. Embolic projection (EP) spine-like, covered by the membranous part of intercalary sclerite. Embolus (Em) with a membranous apex directed posteriorly.

**Female.** Habitus as in Fig. 8A, B. Total length 5.49. Carapace: 2.39 long, 1.85 wide. Eye sizes and interdistances: AME 0.07, ALE 0.09, PME 0.07, PLE 0.08, AME–AME 0.06, AME–ALE 0.03, PME–PME 0.06, PME–PLE 0.06, AME–PME 0.1, AME–PLE 0.13, ALE–ALE 0.23, PLE–PLE 0.32, ALE–PLE 0.06. MOA 0.21 long, front width 0.19, back width 0.21. Chelicera with 3 promarginal and 4 retromarginal teeth. Abdomen: 3.0 long, 1.65 wide. Leg measurements: I 6.18 (1.63, 0.96, 1.34, 1.2, 1.05); II 3.29 (0.95, 0.47, 0.68, 0.62, 0.57); III 4.7 (1.28, 0.56, 0.99, 1.04, 0.83); IV 4.91 (1.37, 0.75, 0.94, 1.03, 0.82). Leg spination: I Fe: d3; Pa: r1; Ti: v1; Mt: v4; II Fe: d2; Pa: r1; Ti: v1; Mt: v4; III Fe: d2, p2, r2; Pa: r1; Ti: p3, r2, v6; Mt: d2, p3, r3; IV: Fe: d2, p2, r2; Pa: r1; Ti: p3, r2, v6; Mt: d1, p4, r4, v2.

Colouration as in male, but paler.

Epigyne (Fig. 8C, D). Epigyne 1.3× longer than wide. Anterior pockets (AP) wavy, longer than median pockets (MP). Posterior pockets (PP) poorly visible. Anterior part of fovea (Fo) subrectangular, 1.5× wider than long. Posterior part of the fovea teardrop-like. Copulatory openings (CO) slit-like, located antero-laterally. Copulatory ducts (CD) consist of three parts: longitudinal anterior part, C-shaped median part, which is 2× wider than anterior, and swollen posterior part. Lateral glands (LG) twice shorter than median part of copulatory ducts, directed laterally. Spermathecae (Sp) oval, connected with copulatory ducts by a short bending tube. Fertilisation ducts (FD) slightly curved.

##### Comments.

The holotype male and the paratype female were collected in different localities. Distance between Guizhumao parking lot and Sanbaishan National Forest Park is about 12 km, which is very close. Moreover, both specimens have similar habitus and coloration. For these reasons, we consider them as conspecific. This hypothesis will be confirmed or rejected in future when both sexes are collected together.

##### Distribution.

**Figure 9.**
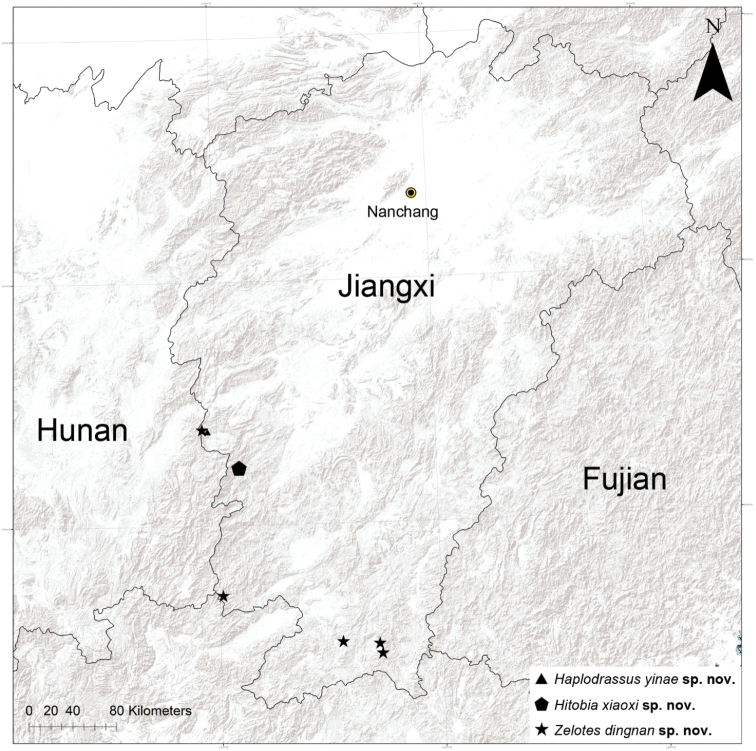
Records of *Haplodrassusyinae* sp. nov., *Hitobiaxiaoxi* sp. nov., and *Zelotesdingnan* sp. nov. from Jiangxi Province, China

Known only from the type locality, Jiangxi Province, China (Fig. 9).

## Supplementary Material

XML Treatment for Haplodrassus

XML Treatment for Haplodrassus yinae

XML Treatment for Hitobia

XML Treatment for Hitobia xiaoxi

XML Treatment for Zelotes

XML Treatment for Zelotes dingnan
